# Association of eating behavior with symptoms of pelvic floor disorders in middle-aged women: An observational study

**DOI:** 10.1177/17455057241305075

**Published:** 2024-12-10

**Authors:** Mari A Kuutti, Matti Hyvärinen, Hannamari Lankila, Pauliina Aukee, Enni-Maria Hietavala, Eija K Laakkonen

**Affiliations:** 1 Gerontology Research Center and Faculty of Sport and Health Sciences, University of Jyväskylä, Jyväskylä, Finland; 2 Department of Obstetrics and Gynecology, Wellbeing Services County of Central Finland, Jyväskylä, Finland

**Keywords:** eating style, pelvic floor function, menopausal women, disordered eating

## Abstract

**Background:**

Estrogen deficiency during menopause, aging, reproductive history, and factors increasing intra-abdominal pressure may lead to structural and functional failure in the pelvic floor. Lifestyle choices, such as eating behavior, may contribute to pelvic floor disorders.

**Objectives:**

The objective of the study was to investigate associations of eating behavior with symptoms of pelvic floor disorders, that is, stress urinary incontinence, urgency urinary incontinence, fecal incontinence, and constipation or defecation difficulties among middle-aged women.

**Design:**

A cross-sectional, observational study was performed using a population sample of 1098 Finnish women aged 47–55 years.

**Methods:**

Eating behavior, food consumption frequency, demographical, gynecological, and physical activity variables were assessed using self-report questionnaires. Logistic regression models were used to assess the associations of eating behavior, food frequency, and symptoms of pelvic floor disorders. Models were adjusted with demographical, gynecological, and physical activity variables.

**Results:**

In adjusted models, middle-aged women with disordered eating style were more likely to experience the symptoms of stress urinary incontinence (odds ratio (OR) 1.5, *p* = 0.002), and constipation or defecation difficulties (OR 1.4, *p* = 0.041). Adding body mass index into the models abolished associations. Of the studied food items, more frequent consumption of ready-made, highly processed foods (OR 1.5, *p* = 0.001), and fast foods (OR 1.5, *p* = 0.005) were independently associated with symptoms of stress urinary incontinence regardless of eating style, whereas consuming ready-made foods (OR 1.4, *p* = 0.048) was associated with symptoms of urgency urinary incontinence. Daily consumption of fruits (OR 0.8, *p* = 0.034) was independently associated with symptoms of stress urinary incontinence. Furthermore, we observed that daily consumption of porridge was associated with symptoms of constipation or defecation difficulties (OR 1.7, *p* = 0.010) independently of eating style. Alcohol consumption (OR 0.9, *p* = 0.015) was inversely associated with constipation and defecation difficulties. Women with overall higher quality diet had lower odds for stress urinary incontinence (OR 0.9, *p* = 0.002).

**Conclusion:**

This study provides proof-of-concept evidence to the hypothesis that eating behavior and consuming certain food items are associated with perceived pelvic floor disorders. As a preventive action, eating behavior of women with the risk of these symptoms should be assessed, and guidance toward healthy eating patterns should be provided.

## Introduction

Pelvic floor disorders in women include conditions that compromise urinary and fecal continence and storage mechanisms.^1–3^ Urinary incontinence alone affects 25%‒45% of women worldwide.^4–7^ Estimated prevalence of fecal incontinence in community dwelling women is approximately 7%–12%,^2,8,9^ whereas prevalence of constipation is reported to be 14%‒16% in the adult population, being more common in women.^10,11^ Embarrassment and lack of knowledge may lead to underreporting the symptoms of pelvic floor disorders. Overall, the disorders negatively affect the quality of life of women of all ages^12^ and cause notable burden to healthcare system.^13^

Estrogen deprivation during menopause, natural aging, reproductive history, and factors increasing intra-abdominal pressure may lead to structural and functional failure in the pelvic floor.^1,14^ In addition, lifestyle choices such as quality of nutrition and eating behavior may have a significant effect on the mechanisms of pelvic floor disorders.^14^ Some macro- and micronutrients, such as protein,^15^ vitamin D,^16^ and omega-3 fatty acids,^17^ are important for proper skeletal muscle function. Disordered eating may result in a lack of these important nutrients; therefore, it may weaken skeletal muscles, including the pelvic floor muscles.^18^ It has been hypothesized that low-energy availability is associated with the development of pelvic floor disorders,^19^ while obesity is also a well-established risk factor.^20–23^ When studying middle-aged women, it is also worth noting that menopausal status might be a specific factor associated with eating behavior.^24^ Estrogens have an important effect on normal food intake, and therefore, they may have a functional role in disordered eating.^25–27^

It is recognized in clinical practice that some dietary items may predispose or exacerbate or, in contrary, relieve the symptoms of pelvic floor disorders. Most previous studies have focused on the effect of a single diet factor, but little is known about the effects of eating behavior in a broader sense. Therefore, further studies related to the association of symptoms of pelvic floor disorders with different aspects of eating behavior are warranted. The objective of the current study is to investigate associations of eating styles with perceived symptoms of pelvic floor disorders, including stress urinary incontinence, urgency urinary incontinence, fecal incontinence, and constipation or defecation difficulties among middle-aged women. In addition to eating styles, the possible associations of the consumption of potential predisposing and protecting dietary items with pelvic floor symptoms are studied. We hypothesized that some eating styles and dietary choices, such a high caffeine intake and low fiber intake, may be associated with a higher risk for the symptoms of pelvic floor disorders, especially urinary incontinence, and constipation or defecation difficulties. In addition, eating styles that are likely to predispose to overweight may concomitantly predispose to the symptoms of pelvic floor disorders. A normal eating style with healthy dietary choices has a beneficial effect on overall health; thereby, it may be protective against the symptoms of pelvic floor disorders.

## Methods

### Study design and participants

The data reported in this secondary analysis are from the cross-sectional, observational study, Estrogenic Regulation of Muscle Apoptosis.^28^ The study data collection was implemented between January 2015 and November 2016 and has been described in detail in Kovanen et al.^29^ Briefly, out of the 6878 randomly selected women aged 47–55 years living in Central Finland, 3064 women returned written consent and prequestionnaire that included questions on symptoms of pelvic floor disorders. Exclusion criteria included conditions or use of medications affecting ovarian function, self-reported body mass index (BMI) >35 kg/m^2^, and medications or symptomatic diseases affecting muscle functions. From the eligible participants, 1393 gave fasting blood samples and 1102 of them answered the main questionnaire survey that included eating behavior questions. Four questionnaires were lost due to technical errors. Therefore, the final analytical sample size of the present study is 1098. This study is reported in accordance with the STROBE (Strengthening the Reporting of Observational Studies in Epidemiology) guidelines.^30^

### Pelvic floor disorders

Participating women were asked to provide a dichotomized response (yes/no) to a questionnaire assessing if they had experienced symptoms of pelvic floor disorders within a month preceding data collection (Supplemental Appendix 1). The questionnaire used is not validated as we chose to use a simplistic questionnaire that is commonly used in clinics in Finland. The symptoms of pelvic floor disorders included in the questionnaire were stress urinary incontinence, urgency urinary incontinence, fecal incontinence, and constipation or defecation difficulties,^31^ and they were studied with the following questions: Have you had within the last month urinary incontinence during physical effort or coughing? Have you had within the last month urge or urgency-related urinary incontinence? Have you had within the last month fecal incontinence? Have you had within the last month constipation or defecation difficulties?

### Eating behavior

*Restrictive eating and overeating* were assessed with the question^32^ “Which of the following best describes you?” and participants were asked to select one of the following four options: “It’s easy for me to eat about the amount I need to”; “I quite often eat more than I actually need”; “I often try to restrict my eating,” and “At times, I’m on a strict diet, at others I overeat.” Following the original publication,^32^ we named these eating styles as normal eating, overeating, restrictive eating, and alternating overeating and restricting, respectively. The crosstabulation between eating styles and symptoms of pelvic floor disorders is presented in the Supplemental Appendix 2. Due to the small sample sizes in groups that included disordered eating patterns, for the regression models, we used normal eating style as a reference class and combined the other eating styles to form a class of disordered eating.

The frequency of consuming drinks and food items was assessed with a 45-item food frequency questionnaire with 6 answering options to choose from: “never or less than once a month,” “once or twice a month,” “once a week,” “twice a week,” “almost every day,” and “once a day or more often.”^33^ Possible predisposing drinks and food items were studied as follows: Consumed coffee was calculated dl/day and alcohol g/week. For the analysis, sugared beverages, low-calorie beverages, ready-made foods (highly processed foods), fast foods, and meat dishes were classified into two categories according to the frequency of consuming the items: rarely, and monthly/weekly. Rarely included “never or less than once a month” answers and monthly/weekly included all the other more frequent answering options. Potential protecting food items, high-fiber bread, porridge, fresh vegetables, and fruits, were classified into categories of monthly/weekly, and daily. Daily included “once a day or more often” answers and monthly/weekly all the other less frequent answering options. The analyzed potential predisposing and protecting drinks and food items were chosen based on the previous literature. Overall diet quality was assessed with diet quality sum score (DQS), which was calculated based on a food frequency questionnaire, as detailed previously by Juppi et al.^34^ Shortly, the questionnaire included 45 typical Finnish food items and 6 answer options for each item. The DQS consisted of 11 food items characteristic of a healthy diet.^35^ Each of the food items was worth 1 point, maximum score being 11 points. A higher score indicates a healthier diet. DQS was constructed based on a previously validated tool.^33^ Slight modifications in the original DQS were necessary because of some minor differences in food items and wordings between the food frequency questionnaires.

### Demographical, physical activity, and gynecological variables

Participants’ demographical, gynecological, and physical activity variables have been described in detail previously.^31^ Shortly, age was calculated from the date of birth to the date of answering to the prequestionnaire. BMI was calculated as body mass (kg) divided by height squared (m^2^). Self-reported education was assessed with a structured question and reclassified into two groups: those with bachelor level or higher education and those with education lower than bachelor level. Four-level physical workload was reclassified into the following groups: mainly sedentary work, work that includes standing and walking, and heavy work that additionally includes lifting.

Physical activity at the age of 17–29 years was assessed with the question: “What kind of regular physical activity have you done at different stages of your life?”^36^ Participants were asked to specify their participation by selecting one or more of the following four options: no physical activity, regular independent leisure-time physical activity, regular competitive sport and related training, and regular other supervised physical activity in a sports club, etc. Current physical activity was evaluated with a self-reported questionnaire^37^ including four questions about the frequency, intensity, and duration of leisure-time physical activity bouts as well as the average time spent in active commuting. Based on the answers, a metabolic equivalent of hours per day for current physical activity was calculated.

Participants were assigned to premenopausal, early and late perimenopausal, and postmenopausal groups based on the follicle stimulating hormone concentrations and self-reported menstrual bleeding diaries using the slightly modified Stages of Reproductive Aging Workshop (STRAW+10) guidelines.^38^ Self-reported data on parity and whether a participant had undergone hysterectomy were collected.

### Missing data

Of the analytical sample including 1098 participants, 2 had missing data on eating styles and 4–8 on different pelvic floor symptoms (Supplemental Appendix 1). The total number of missing data values was 287 out of 29,646 datapoints (1.0%). The percentage of missing values varied from 0% to 10% between the variables. The data was missing due to invalid or missing measurements and unclear or incomplete questionnaire responses. Thus, missing data were assumed to occur at random. Multiple imputation was used to create and analyze 50 multiply imputed data sets with 50 iterations for chained equations for each.^39^ The model parameters were estimated separately for each data set. Multiple imputation and pooling of the model estimates were carried out in R^40^ using the standard settings of the “mice” package.^39^ For comparison, we also performed a complete case analysis, resulting in no major differences in the model estimates.

### Statistical analyses

The associations of eating styles with symptoms of pelvic floor disorders were analyzed with four different models (Table 3). Model 1 was a simple logistic regression model and model 2 was adjusted with age and menopausal status. Model 3 was additionally adjusted with physical workload, physical activity history, current physical activity, and parity. For the final model (model 4), BMI was added to the list of confounders. The confounders with potential effects on the predictor and outcome variable identified based on the literature were included in the models. BMI was included individually in the fourth model to study its potentially significant role in the association between eating styles and symptoms of pelvic floor disorders.

The associations of potential predisposing (Tables 4 and 5) and protecting food items (Table 6) with symptoms of pelvic floor disorders were analyzed with two different models. Model 1 was a logistic regression model adjusted with age and menopausal status and model 2 was additionally adjusted with eating style. To allow robust investigation of the role of food items in different eating style backgrounds we decided to limit our attempts to control for confounding bias to the most obvious confounders, age, and menopausal status.

Correlation analysis, residual plots and scatter plots between each continuous predictor and the logits values were used for testing the model assumptions. Statistical analyses were performed using R and IBM SPSS Statistics 22.0 (SPSS Inc., Chicago, IL, USA). The level of significance was set at *p* ⩽ 0.05.

## Results

Supplemental Appendix 2 shows the prevalence of the symptoms of pelvic floor disorders in the total analytical sample and across the four different eating styles. Participants’ self-reported symptoms of pelvic floor disorders, gynecological variables, physical activity status as well as demographical descriptives in total analytical sample and in normal and disordered eating styles are presented in Table 1. All symptoms of pelvic floor disorders were more common in women with disordered eating style compared to women with normal eating style. Women with disordered eating style had undergone hysterectomy more often, were currently less physically active, had lower education, and had notably higher BMI than women who had reported normal eating style. Average age of the participants was 51.2 years. The frequencies of consuming different drinks and food items in total analytical sample and in normal and disordered eating styles are presented in Table 2.

**Table 1.** Descriptive data in different eating styles (*n* = 1098).

Variables	Total analytical sample (*n* = 1098)	Normal eating (*n* = 589)^a^	Disordered eating (*n* = 507)
Pelvic floor disorders, *n* (%)			
Stress urinary incontinence	440 (40.3)	208 (35.5)	231 (45.8)
Missing data, *n*	6	3	3
Urgency urinary incontinence	149 (13.6)	76 (12.9)	73 (14.5)
Missing data, *n*	6	2	4
Fecal incontinence	34 (3.1)	13 (2.2)	21 (4.2)
Missing data, *n*	8	3	5
Constipation or defecation difficulties	189 (17.3)	88 (15.0)	101 (20.1)
Missing data, *n*	6	2	4
Gynecological variables			
Menopausal status, *n* (%)			
Premenopause	304 (27.7)	161 (27.3)	143 (28.2)
Early perimenopause	198 (18.0)	101 (17.1)	97 (19.1)
Late perimenopause	209 (19.0)	108 (18.3)	101 (19.9)
Postmenopause	387 (35.2)	219 (37.2)	166 (32.7)
Missing data, *n*	0	0	0
Parity, mean (SD)	2.0 (1.2)	2.0 (1.2)	2.0 (1.3)
Missing data, *n*	2	0	2
Hysterectomy, *n* (%)			
Yes	90 (8.2)	34 (5.8)	56 (11.1)
No	1007 (91.8)	555 (94.2)	450 (88.9)
Missing data, *n*	1	0	1
Physical activity			
Physical workload, *n* (%)			
Light	535 (53.0)	277 (52.0)	257 (54.1)
Moderate	203 (20.1)	120 (22.5)	83 (17.5)
Heavy	271 (26.9)	136 (25.5)	135 (28.4)
Missing data, *n*	89	56	32
Previous PA (age 17–29), *n* (%)			
No exercise	234 (23.7)	110 (21.0)	124 (27.0)
Regular PA	658 (66.7)	363 (69.1)	294 (63.9)
Competitive sport	94 (9.5)	52 (9.9)	42 (9.1)
Missing data, *n*	112	64	47
Current PA (MET-h/d), mean (SD)	3.7 (3.7)	4.1 (4.0)	3.2 (3.3)
Missing data, *n*	6	5	1
Demographical descriptives			
Age, mean (SD)	51.2 (2.0)	51.2 (2.1)	51.2 (2.0)
Missing data, *n*	0	0	0
Education, *n* (%)			
Secondary	643 (58.6)	329 (55.9)	313 (61.7)
Tertiary	455 (41.4)	260 (44.1)	194 (38.3)
Missing data, *n*	0	0	0
Body mass index, mean (SD)	25.2 (3.5)	23.7 (2.9)	27.1 (3.3)
Missing data, *n*	5	1	4

SD: standard deviation; MET-h/d: metabolic equivalent hours per day; PA: physical activity.

a Two women did not report their eating style.

**Table 2.** Consumption of food items in different eating styles (*n* = 1098).

Food items	Total analytical sample (*n* = 1098)	Normal eating (*n* = 589)^a^	Disordered eating (*n* = 507)
Diet quality score, mean (SD)	5.8 (2.2)	6.1 (2.2)	5.4 (2.2)
Missing data, *n*	8	6	2
Coffee (dl/day), mean (SD)	3.7 (2.5)	3.6 (2.4)	4.0 (2.6)
Missing data, *n*	0	0	0
Alcohol (g/week), mean (SD)	3.8 (3.8)	3.5 (3.7)	4.2 (3.8)
Missing data, *n*	0	0	0
Sugared beverages			
Rarely	743 (67.8)	411 (69.9)	330 (65.2)
Monthly/weekly	353 (32.2)	177 (30.1)	176 (34.8)
Missing data, *n*	2	1	1
Low-calorie beverages			
Rarely	835 (76.0)	483 (82.0)	350 (69.0)
Monthly/weekly	263 (24.0)	106 (18.0)	157 (31.0)
Missing data, *n*	0	0	0
Ready-made foods (highly processed foods)			
Rarely	595 (54.5)	336 (57.6)	257 (50.7)
Monthly/weekly	497 (45.5)	247 (42.4)	250 (49.3)
Missing data, *n*	6	6	0
Fast foods			
Rarely	837 (76.7)	462 (79.0)	373 (74.0)
Monthly/weekly	254 (23.3)	123 (21.0)	131 (26.0)
Missing data, *n*	7	4	3
Meat dishes			
Rarely	88 (8.1)	47 (8.0)	41 (8.1)
Monthly/weekly	1002 (91.9)	537 (92.0)	468 (91.9)
Missing data, *n*	8	5	3
High-fiber bread			
Monthly/weekly	495 (45.2)	254 (43.3)	240 (47.3)
Daily	601 (54.8)	333 (56.7)	267 (52.7)
Missing data, *n*	2	2	0
Porridge			
Monthly/weekly	925 (84.5)	480 (81.9)	443 (87.4)
Daily	170 (15.5)	106 (18.1)	64 (12.6)
Missing data, *n*	3	3	0
Fresh vegetables			
Monthly/weekly	523 (47.7)	245 (41.7)	278 (54.9)
Daily	573 (52.3)	343 (58.3)	228 (45.1)
Missing data, *n*	2	1	1
Fruits			
Monthly/weekly	653 (59.6)	336 (57.2)	316 (62.3)
Daily	443 (40.4)	251 (42.8)	191 (37.7)
Missing data, *n*	2	2	0

a Two women did not report their eating style.

Women with disordered eating had a higher risk for stress urinary incontinence in unadjusted model 1 (odds ratio (OR) 1.54, *p* = 0.001) as well as in adjusted model 2 (OR 1.54, *p* = 0.001) and model 3 (OR 1.49, *p* = 0.002) (Table 3). Similarly, disordered eating was associated with constipation or defecation difficulties according to model 1 (OR 1.42, *p* = 0.028), model 2 (OR 1.41, *p* = 0.034) and model 3 (OR 1.40, *p* = 0.041). However, the statistical significance of the associations was abolished after additionally adjusting for BMI in Model 4.

**Table 3.** Associations of eating styles with pelvic floor disorders (*n* = 1098).

Eating styles	Stress urinary incontinence		Urgency urinary incontinence		Fecal incontinence		Constipation or defecation difficulties	
	OR (95% CI)	*p-*Value	OR (95% CI)	*p-*Value	OR (95% CI)	*p-*Value	OR (95% CI)	*p-*Value
Model 1								
Normal eating (ref)	1		1		1		1	
Disordered eating	1.54 (1.21–1.96)	**0.001**	1.14 (0.81–1.62)	0.447	1.96 (0.97–3.96)	0.060	1.42 (1.04–1.95)	**0.028**
Model 2								
Disordered eating	1.54 (1.20–1.96)	**0.001**	1.15 (0.82–1.63)	0.419	1.98 (0.98–4.01)	0.058	1.41 (1.03–1.92)	**0.034**
Model 3								
Disordered eating	1.49 (1.16–1.92)	**0.002**	1.16 (0.81–1.65)	0.416	2.01 (0.99–4.12)	0.055	1.40 (1.01–1.93)	**0.041**
Model 4								
Disordered eating	1.13 (0.85–1.49)	0.414	1.08 (0.73–1.61)	0.707	1.45 (0.65–3.22)	0.360	1.29 (0.90–1.85)	0.158

Model 1: Simple logistic regression.

Model 2: Adjusted with age and menopausal status.

Model 3: Model 2+physical workload, physical activity (age 17–29), current physical activity (MET-h/d), and parity.

Model 4: Model 3+body mass index.

MET-h/d: metabolic equivalent hours per day; OR: odds ratio; 95% CI: 95% confidence interval.

Bold values indicate significant at *p* < 0.05.

Alcohol consumption was inversely associated with constipation and defecation difficulties in model 1 (OR 0.95, *p* = 0.026) and in model 2 (OR 0.94, *p* = 0.015) (Table 4). Higher consuming frequency of ready-made foods was associated with higher risks for stress urinary incontinence in model 1 (OR 1.53, *p* = <0.001) and in model 2 (OR 1.50, *p* = 0.001) as well as with urgency urinary incontinence in model 1 (OR 1.43, *p* = 0.042) and in model 2 (OR 1.42, *p* = 0.048) (Table 5). Similarly, eating fast foods more often was associated with higher risk for stress urinary incontinence according to both model 1 (OR 1.54, *p* = 0.003) and model 2 (OR 1.51, *p* = 0.005).

**Table 4.** Potential predisposing drinks in different pelvic floor disorders (*n* = 1098).

Drinks	Stress urinary incontinence		Urgency urinary incontinence		Fecal incontinence		Constipation or defecation difficulties	
	OR (95% CI)	*p-*Value	OR (95% CI)	*p-*Value	OR (95% CI)	*p-*Value	OR (95% CI)	*p-*Value
Model 1								
Coffee	1.04 (0.99–1.09)	0.117	1.04 (0.97–1.11)	0.308	1.10 (0.97–1.24)	0.137	0.98 (0.91–1.04)	0.465
Model 2								
Coffee	1.03 (0.98–1.09)	0.193	1.03 (0.97–1.11)	0.338	1.09 (0.96–1.24)	0.184	0.97 (0.91–1.04)	0.366
Disordered eating style (ref. normal)	1.52 (1.19–1.94)	**<0.001**	1.13 (0.80–1.61)	0.470	1.86 (0.92–3.76)	0.084	1.44 (1.05–1.97)	**0.025**
Model 1								
Alcohol	0.99 (0.95–1.02)	0.456	0.98 (0.93–1.03)	0.400	1.00 (0.91–1.09)	0.957	0.95 (0.90–0.99)	**0.026**
Model 2								
Alcohol	0.98 (0.95–1.01)	0.298	0.98 (0.93–1.03)	0.363	0.99 (0.90–1.09)	0.834	0.94 (0.89–0.99)	**0.015**
Disordered eating style (ref. normal)	1.56 (1.22–1.99)	**<0.001**	1.17 (0.83–1.65)	0.379	1.94 (0.96–3.93)	0.064	1.48 (1.08–2.03)	**0.016**
Model 1								
Monthly/weekly sugared beverages (ref. rarely)	1.15 (0.89–1.49)	0.294	1.00 (0.69–1.45)	0.981	0.86 (0.41–1.83)	0.703	1.22 (0.88–1.69)	0.242
Model 2								
Monthly/weekly sugared beverages (ref. rarely)	1.12 (0.87–1.46)	0.377	1.00 (0.69–1.44)	0.988	0.83 (0.39–1.77)	0.637	1.20 (0.86–1.66)	0.315
Disordered eating style (ref. normal)	1.53 (1.20–1.96)	**<0.001**	1.15 (0.81–1.63)	0.423	1.95 (0.96–3.93)	0.063	1.41 (1.03–1.93)	**0.045**
Model 1								
Monthly/weekly low-calorie beverages (ref. rarely)	0.96 (0.72–1.27)	0.768	0.77 (0.50–1.18)	0.227	1.33 (0.63–2.82)	0.456	1.00 (0.69–1.44)	0.991
Model 2								
Monthly/weekly low-calorie beverages (ref. rarely)	0.88 (0.66–1.18)	0.404	0.74 (0.48–1.15)	0.181	1.19 (0.56–2.55)	0.648	0.94 (0.64–1.36)	0.731
Disordered eating style (ref. normal)	1.57 (1.22–2.00)	**<0.001**	1.19 (0.84–1.69)	0.319	1.89 (0.93–3.83)	0.080	1.42 (1.04–1.97)	**0.028**

Model 1: Adjusted with age and menopausal status.

Model 2: Model 1+adjusted with eating style.

OR: odds ratio; 95% CI: 95% confidence interval.

Bold values indicate significant at *p* < 0.05.

**Table 5.** Potential predisposing food items in different pelvic floor disorders (*n* = 1098).

Food items	Stress urinary incontinence		Urgency urinary incontinence		Fecal incontinence		Constipation or defecation difficulties	
	OR (95% CI)	*p-*Value	OR (95% CI)	*p-*Value	OR (95% CI)	*p-*Value	OR (95% CI)	*p-*Value
Model 1								
Monthly/weekly ready-made foods (highly processed foods) (ref. rarely)	1.53 (1.20–1.96)	**<0.001**	1.43 (1.01–2.03)	**0.042**	1.16 (0.58–2.30)	0.678	1.22 (0.89–1.68)	0.213
Model 2								
Monthly/weekly ready-made foods (highly processed foods) (ref. rarely)	1.50 (1.17–1.92)	**0.001**	1.42 (1.00–2.02)	**0.048**	1.11 (0.55–2.21)	0.772	1.19 (0.87–1.64)	0.271
Disordered eating style (ref. normal)	1.50 (1.18–1.92)	**0.001**	1.12 (0.79–1.59)	0.507	1.91 (0.95–3.87)	0.070	1.40 (1.02–1.24)	**0.036**
Model 1								
Monthly/weekly fast foods (ref. rarely)	1.54 (1.16–2.05)	**0.003**	0.91 (0.60–1.38)	0.653	0.99 (0.44–2.23)	0.990	0.75 (0.51–1.12)	0.161
Model 2								
Monthly/weekly fast foods (ref. rarely)	1.51 (1.13–2.00)	**0.005**	0.90 (0.59–1.37)	0.619	0.95 (0.42–2.14)	0.902	0.73 (0.49–1.09)	0.127
Disordered eating style (ref. normal)	1.51 (1.18–1.93)	**<0.001**	1.16 (0.82–1.64)	0.407	1.94 (0.96–3.91)	0.065	1.44 (1.05–1.98)	**0.023**
Model 1								
Monthly/weekly meat dishes (ref. rarely)	1.47 (0.92–2.35)	0.106	1.23 (0.62–2.45)	0.545	1.22 (0.29–5.09)	0.783	1.33 (0.71–2.51)	0.371
Model 2								
Monthly/weekly meat dishes (ref. rarely)	1.48 (0.92–2.37)	0.103	1.24 (0.62–2.45)	0.544	1.23 (0.29–5.11)	0.780	1.34 (0.71–2.52)	0.368
Disordered eating style (ref. normal)	1.54 (1.21–1.97)	**<0.001**	1.15 (0.82–1.63)	0.422	1.93 (0.96–3.89)	0.066	1.42 (1.04–1.95)	**0.029**

Model 1: Adjusted with age and menopausal status.

Model 2: Model 1+adjusted with eating style.

OR: odds ratio; 95% CI: 95% confidence interval.

Bold values indicate significant at *p* < 0.05.

Consuming fruits more often was associated with lower risk for stress urinary incontinence according to model 1 (OR 0.75, *p* = 0.022) and model 2 (OR 0.76, *p* = 0.034) (Table 6). Consuming porridge more often was associated with higher risk for constipation or defecation difficulties according to model 1 (OR 1.62, *p* = 0.018) and model 2 (OR 1.69, *p* = 0.010). Women with overall higher quality diet had lower odds for stress urinary incontinence according to model 1 (OR 0.90, *p* = <0.001) and model 2 (OR 0.91, *p* = 0.002).

**Table 6.** Potential protecting food items and diet quality score in different pelvic floor disorders (*n* = 1098).

Food items	Stress urinary Incontinence		Urgency urinary incontinence		Fecal incontinence		Constipation or defecation difficulties	
	OR (95% CI)	*p-*Value	OR (95% CI)	*p-*Value	OR (95% CI)	*p-*Value	OR (95% CI)	*p-*Value
Model 1								
Daily high-fiber bread (ref. monthly/weekly)	1.01 (0.79–1.29)	0.950	0.86 (0.61–1.22)	0.393	1.76 (0.85–3.65)	0.127	0.99 (0.72–1.35)	0.930
Model 2								
Daily high-fiber bread (ref. monthly/weekly)	1.03 (0.80–1.31)	0.837	0.86 (0.61–1.22)	0.411	1.82 (0.87–3.77)	0.109	1.00 (0.73–1.37)	0.999
Disordered eating style (ref. normal)	1.54 (1.21–1.97)	**<0.001**	1.14 (0.81–1.62)	0.442	1.98 (0.98–4.00)	0.057	1.42 (1.04–1.94)	**0.029**
Model 1								
Daily porridge (ref. monthly/weekly)	0.76 (0.54–1.07)	0.119	0.99 (0.62–1.60)	0.979	0.75 (0.26–2.16)	0.599	1.62 (1.09–2.40)	**0.018**
Model 2								
Daily porridge (ref. monthly/weekly)	0.79 (0.56–1.12)	0.188	1.01 (0.62–1.63)	0.973	0.81 (0.28–2.32)	0.688	1.69 (1.13–2.52)	**0.010**
Disordered eating style (ref. normal)	1.52 (1.19–1.94)	**<0.001**	1.15 (0.81–1.63)	0.423	1.91 (0.95–3.86)	0.071	1.47 (1.07–2.02)	**0.018**
Model 1								
Daily fresh vegetables (ref. monthly/weekly)	0.82 (0.64–1.04)	0.105	0.80 (0.57–1.13)	0.209	0.62 (0.31–1.36)	0.272	0.76 (0.55–1.04)	0.086
Model 2								
Daily fresh vegetables (ref. monthly/weekly)	0.86 (0.68–1.10)	0.241	0.81 (0.57–1.15)	0.246	0.68 (0.34–1.36)	0.271	0.79 (0.57–1.09)	0.150
Disordered eating style (ref. normal)	1.51 (1.18–1.93)	**0.001**	1.12 (0.79–1.59)	0.523	1.83 (0.90–3.72)	0.093	1.38 (1.00–1.89)	**0.049**
Model 1								
Daily fruits (ref. monthly/weekly)	0.75 (0.58–0.96)	**0.022**	0.83 (0.58–1.18)	0.288	0.53 (0.24–1.14)	0.105	1.13 (0.83–1.56)	0.439
Model 2								
Daily fruits (ref. monthly/weekly)	0.76 (0.59–0.98)	**0.034**	0.83 (0.58–1.19)	0.307	0.54 (0.25–1.18)	0.125	1.15 (0.84–1.59)	0.372
Disordered eating style (ref. normal)	1.52 (1.19–1.94)	**<0.001**	1.14 (0.81–1.61)	0.455	1.88 (0.93–3.80)	0.079	1.43 (1.04–1.96)	**0.026**
Model 1								
Diet quality score	0.90 (0.85–0.95)	**<0.001**	0.98 (0.90–1.06)	0.550	1.03 (0.88–1.20)	0.749	0.97 (0.90–1.04)	0.369
Model 2								
Diet quality score	0.91 (0.86–0.97)	**0.002**	0.98 (0.91–1.06)	0.635	1.05 (0.90–1.23)	0.530	0.98 (0.91–1.05)	0.579
Disordered eating style (ref. normal)	1.45 (1.13–1.86)	**0.003**	1.14 (0.80–1.61)	0.475	2.00 (0.98–4.08)	0.056	1.40 (1.02–1.93)	**0.039**

Model 1: Adjusted with age and menopausal status.

Model 2: Model 1+adjusted with eating style.

OR: odds ratio; 95% CI: 95% confidence interval.

Bold values indicate significant at *p* < 0.05.

## Discussion

This study examined the association of eating behavior and dietary choices with symptoms of pelvic floor disorders in middle-aged women. We found that women who reported disordered eating were more likely to experience the symptoms of urinary incontinence and constipation or defecation difficulties. In addition, some drinks and dietary items were potentially predisposing or protecting regarding these conditions. Overall, women with higher quality diet had lower odds for symptoms of stress urinary incontinence.

Disordered eating, characterized by maladaptive eating attitudes and behaviors, seem to be common among middle-aged women in Western societies.^41^ The causes might lie in the biological (e.g., BMI and menopausal status), psychological (e.g., aging anxiety), and sociocultural factors (e.g., perceived pressure to be thin).^41,42^ It has been hypothesized that menopausal transition increases vulnerability to eating-related conditions, such as eating disorders and negative body image,^27,43^ and, on the contrary, that disordered eating or body image concerns do not differ between menopausal phases.^44,45^ Mangweth-Matzek et al.^46^ found that menopausal symptomology, as assessed by the Menopause Rating Scale, showed strong associations with eating and body-image measures, whereas defined menopausal stages showed no significant associations. However, there is substantial evidence that reproductive hormones play an important role in eating behavior:^47–51^ In women, the control of food intake is largely regulated by estradiol, which acts as an inhibitor by decreasing meal size and advancing satiety.^47–49,52^

Restrained eating or dieting refers to the intentional and sustained restriction of food intake for the purposes of weight loss or weight maintenance.^53,54^ Restrained eating appears to be relatively common behavior among middle-aged women.^24^ In the study of Drobnjak et al.,^24^ 10.7% of normal-weight women aged between 40 and 66 reported to engage in extreme dietary restraint. The authors described that postmenopausal women reported higher levels of restrained eating compared to premenopausal women. Another study^55^ examined overweight middle-aged women and showed also increased restrained eating after menopause. The present study is in line with the previous, since postmenopausal women reported restricting their eating more than pre- and perimenopausal women. Overall, 10.6% of all participating women reported to restrict their eating.

Another form of disordered eating is overconsumption of palatable food, which can appear in the form of compulsive overeating or bingeing.^56^ Generally, it involves eating more than an individual currently physiologically needs.^57^ Differences in eating disorder symptoms and symptom pathways across developmental stages were examined in a large-scale study by Christian et al.^58^ Findings suggested that older women, including women in midlife, may experience binge eating symptoms more than women in adolescence and early young adulthood. Another study on the subject resulted in contradictory findings: In a longitudinal study of Brown et al.,^59^ women were followed from young adulthood through midlife and changes in their eating disorder diagnoses and symptoms were examined. The study found significantly fewer eating disorder diagnoses and binge eating symptoms during midlife (ages 48–52) as compared to baseline (ages 18–22). In our sample of middle-aged women, 32.5% reported to overeat frequently. Postmenopausal women reported to overeat more than pre- and perimenopausal women.

We found that women who had reported to have features of disordered eating were more likely to experience the symptoms of stress urinary incontinence than women with normal eating style. The association between diet and the onset of stress urinary incontinence was assessed in the longitudinal Leicester MRC Incontinence Study^60^ with over 5800 women aged 40 years plus. Higher total fat intake was significantly associated with stress urinary incontinence. In particular, saturated fatty acids (SFA) and cholesterol increased the risk for symptoms. It has also been shown that the higher total daily calorie intake is associated with urinary incontinence and increased severity.^61^ Likewise, ratio of SFA intake to polyunsaturated fat acid (PUFA) intake is positively associated with urinary incontinence and its severity. In the study of Maserejian et al.,^61^ women had over twice the odds of urinary incontinence if their SFA:PUFA ratio was greater than 2:1. Ready-made, highly processed foods, and fast foods often contain higher levels of fat, especially saturated fat. These features of diet are linked to obesity and higher BMI as well as the risk of pelvic floor disorders.^60,62,63^ However, the causal relationship between eating behavior, BMI, and pelvic floor disorder is not clear warranting further studies. In our sample of women with an average BMI of 25.2, indicating slight overweight, the associations between disordered eating and pelvic floor disorders seemed to be largely explained by the BMI. After adjusting our models with BMI, the associations became smaller and not statistically significant. Albeit our study did not investigate body composition or location of body fat, the plausible explanation for the confounding role of the BMI is that a woman’s higher BMI indicates higher central body adiposity, which might bear down on pelvic tissues causing chronic strain, stretching, and weakening of the structures of the pelvic floor.^64^

The analysis of potential dietary risk factors shows that consuming monthly or more often ready-made and fast foods leads to a higher risk for symptoms of stress urinary incontinence. The same applies with ready-made foods and urgency urinary incontinence. The mechanism behind this phenomenon may lead to metabolic aspects: Unhealthy Western style diet, characterized by high consumption of saturated fats and overeating, causes oxidative stress, whereas excessive fat consumption contributes to the development of low-grade systemic inflammation.^65^ Oxidative stress and systemic inflammation are associated with both stress and urgency urinary incontinence.^66,67^ The hypothesized role of inflammatory processes in the etiology of urologic symptoms is supported by the results of the BACH survey in which Kupelian et al.^68^ reported that increased serum C-reactive protein increases the odds of lower urinary tract symptoms in women. Fruits are known to contain high amounts of antioxidants, which counteract inflammatory processes in the body.^69^ According to our study, eating fruits daily may protect from the symptoms of stress urinary incontinence. In addition, it is plausible that diet rich in antioxidants may as well be of higher quality in other measures since higher diet quality indicated lower risk for stress urinary incontinence. Previous studies have shown that carbonated drinks, artificial sweeteners, caffeine, and alcohol are bladder irritants.^70–72^ Nevertheless, drinks such as coffee, alcohol, and different types of beverages were not associated with symptoms of urinary incontinence in our sample.

According to the present study, features of disordered eating, including restricted eating, overeating or their combination, are associated with symptoms of constipation or defecation difficulties. It has been shown that overeating individuals tend to choose food items high in fat and carbohydrates and low in fiber.^63^ Inadequate dietary fiber intake is a potential risk factor for constipation, since fiber serves to enhance stool consistency and stimulates bowel movements.^73,74^ In the study of Dukas et al.,^75^ the influence of lifestyle variables, including fiber intake, on the development of constipation was evaluated by the responses of 62036 women (age 36–61 years). A total of 3327 women (5.4%) were classified as having constipation, defined as 2 or fewer bowel movements weekly. Women in the highest quintile of dietary fiber intake (20 g/day) were less likely to experience constipation than women in the lowest quintile (7 g/day). However, a review article^76^ summarized that a diet poor in fiber may be a contributory factor but should not be assumed to be the cause of chronic constipation. Some patients may benefit from a fiber-rich diet, but patients with more severe constipation may get worse symptoms when fiber intake is increased. Interestingly, we found that women with a habit to consume porridge more often are at higher risk for the symptoms of constipation or defecation difficulties. We were not able to study causal effects; thus, it can only be speculated whether reverse causality exists, and women with constipation or defecation difficulties try to relieve the symptoms by eating more fibers. Overall, soluble fiber, found, for example, in oat, may be of benefit in treating chronic constipation.^74^ Inadequate fluid intake is suggested to be a risk factor for constipation; however, there are inconsistencies in study results, and fluid intake may be of benefit only for dehydrated patients.^76^ Previously, daily alcohol consumption (at least one drink; approximately 12 g of alcohol) has been found to be inversely associated with constipation,^75^ a result in line with our study.

We did not find statistically significant associations between symptoms of fecal incontinence and eating style. However, women may modify their eating when trying to control the symptoms. In a qualitative study of Andy et al.,^77^ dietary modification strategies were studied with community-dwelling older women who tried to manage their fecal incontinence. Women were aware of the role of the diet and made dietary modifications including decreasing the amounts and frequency of meals, limiting certain foods (such as coffee, and spicy and fatty foods), and changing methods of food preparation (baking and boiling instead of frying). Others have also reported that certain foods are perceived to improve or exacerbate fecal incontinence symptoms, and diet is adjusted as part of self-care practices.^78,79^

Studies on the role of diet in the pathogenesis of fecal incontinence are scarce. However, it has been shown that macro-and micronutrient intake does not differ significantly in those with and without fecal incontinence.^78^ Dietary fiber may be an exception since low fiber intake has been found to be independently associated with fecal incontinence,^80^ and its adequate intake is broadly advised for bowel health.^81,82^ However, compared to constipation, data on the effects of fiber on fecal incontinence are limited. We studied effects of liquids, low-fiber fatty foods, and high-fiber foods with symptoms of fecal incontinence, finding no associations. Our sample included a rather small number of women experiencing fecal incontinence (34 cases, 3.1%). Therefore, our results regarding fecal incontinence cannot be considered conclusive.

### Strengths and limitations

The present study had several strengths and limitations. We were able to utilize previously gained knowledge in considering potential confounding factors, that is, to control also for past and current physical activity and menopausal status of participants in addition to demographical and gynecological factors.^31^ Overall, the extent of this study is exceptional, since we were able to study four different symptoms of pelvic floor disorders among the large homogenous cohort of Caucasian women. The homogeneity of the study population is simultaneously a strength as it reduces internal variability and increases statistical power and a limitation as our findings may not be reproducible in more heterogeneous populations. It can be considered as a limitation that we have not made *a priori* power calculations, because this is an observational cohort study.

The experienced symptoms of pelvic floor disorders were asked in an early stage of the study, which may result in underreporting, especially when the subject may be considered sensitive. We chose to use a simplistic, short pelvic floor disorder questionnaire that is fast and easy to fill in as part of a self-report questionnaire and is commonly used in clinics as part of medical examination interviews. However, the questionnaire used is not validated, which is a limitation. Since the responses were dichotomized (yes/no), we were not able to study the severity of the symptoms. We were also not able to ensure that women understood the possible symptoms in a similar manner. Women with BMI >35 kg/m^2^ were excluded from the study; thus, the results are not completely generalizable to individuals with severe obesity. In addition, the assessment of eating behavior based on self-reporting can be biased by social desirability,^83^ that is, the tendency to assess one’s own eating styles critically, which affects women more than men.^84^ This may cause respondents to overestimate healthy behaviors and underestimate the undesirable ones.^85,86^ The study was explorative cross-sectional observational study and cannot therefore reveal women’s long-term eating habits and whether they have a causal effect on the development of the symptoms of pelvic floor disorders or if reverse causality exists.

## Conclusions

This study was exploratory in nature. Eating behavior has scantly been studied as a potential risk factor for pelvic floor disorders, and thus, we aimed to test the proof-of-concept. In our sample, women with disordered eating style, including women with overeating, restrictive eating, and alternating overeating and restricting eating styles, were more prone to experience symptoms of pelvic floor disorders compared to women with normal eating style. To our knowledge, there are no previous studies investigating the association of eating style with pelvic floor disorders in the middle-aged female population nor any other aged women. In addition, this study revealed that some dietary items may predispose or protect from the symptoms, providing information for healthcare practitioners and patients themselves. Since we found the disordered eating style and some dietary items to be associated with perceived pelvic floor disorders, our study justifies further studies with longitudinal designs to investigate causality.

## Supplemental Material

sj-docx-1-whe-10.1177_17455057241305075 – Supplemental material for Association of eating behavior with symptoms of pelvic floor disorders in middle-aged women: An observational study Supplemental material, sj-docx-1-whe-10.1177_17455057241305075 for Association of eating behavior with symptoms of pelvic floor disorders in middle-aged women: An observational study by Mari A Kuutti, Matti Hyvärinen, Hannamari Lankila, Pauliina Aukee, Enni-Maria Hietavala and Eija K Laakkonen in Women’s Health

sj-docx-2-whe-10.1177_17455057241305075 – Supplemental material for Association of eating behavior with symptoms of pelvic floor disorders in middle-aged women: An observational study Supplemental material, sj-docx-2-whe-10.1177_17455057241305075 for Association of eating behavior with symptoms of pelvic floor disorders in middle-aged women: An observational study by Mari A Kuutti, Matti Hyvärinen, Hannamari Lankila, Pauliina Aukee, Enni-Maria Hietavala and Eija K Laakkonen in Women’s Health

sj-docx-3-whe-10.1177_17455057241305075 – Supplemental material for Association of eating behavior with symptoms of pelvic floor disorders in middle-aged women: An observational study Supplemental material, sj-docx-3-whe-10.1177_17455057241305075 for Association of eating behavior with symptoms of pelvic floor disorders in middle-aged women: An observational study by Mari A Kuutti, Matti Hyvärinen, Hannamari Lankila, Pauliina Aukee, Enni-Maria Hietavala and Eija K Laakkonen in Women’s Health

## Declarations

**Ethics approval and consent to participate:** The study was conducted according to the Declaration of Helsinki, except that preregistration was not done. The study was approved by the Ethics Committee of the Central Finland Health Care District (KSSHP Dnro 8U/2014) before the onset of data collection. All participants provided written informed consent after being informed about the objectives of the study.

**Consent for publication:** Not applicable.

**Availability of data and materials:** The datasets analyzed during the current study are available in the JYX Digital Repository https://doi.org/10.17011/jyx/dataset/83491.

## References

[1] Johnston SL. Pelvic floor dysfunction in midlife women. Climacteric 2019; 22(3): 270–276. 30857432 10.1080/13697137.2019.1568402

[bibr2-17455057241305075] Melville JL. Fecal incontinence in US women: a population-based study. Am J Obstet Gynecol 2005; 193(6): 2071–2076. 16325618 10.1016/j.ajog.2005.07.018

[3] Bharucha AE. Constipation. Best Pract Res Clin Gastroenterol 2007; 21(4): 709–731. 17643910 10.1016/j.bpg.2007.07.001

[4] Milsom I Altman D Cartwright R , et al. Epidemiology of urinary incontinence (UI) and other lower urinary tract symptoms (LUTS), pelvic organ prolapse (POP) and anal (AI) incontinence. In: Abrams P, Cardozo L, Khoury S and Wein AJ (eds) Incontinence, 6th ed. Paris: Health Publications Ltd, 2016.

[bibr5-17455057241305075] Melville JL Katon W Delaney K , et al. Urinary incontinence in US women: a population-based study. Arch Intern Med 2005; 165(5): 537–542. 15767530 10.1001/archinte.165.5.537

[bibr6-17455057241305075] Zhu L Lang J Wang H , et al. The prevalence of and potential risk factors for female urinary incontinence in Beijing, China. Menopause 2008; 15(3): 566–569. 18467955 10.1097/gme.0b013e31816054ac

[7] Yarnell JW Voyle GJ Richards CJ , et al. The prevalence and severity of urinary incontinence in women. J Epidemiol Community Health 1981; 35(1): 71. 7264536 10.1136/jech.35.1.71 PMC1052123

[8] Aitola P. Prevalence of faecal incontinence in adults aged 30 years or more in general population. Colorectal Dis 2010; 12(7): 687–691. 19486087 10.1111/j.1463-1318.2009.01878.x

[9] Bharucha AE. Prevalence and burden of fecal incontinence: a population-based study in women. Gastroenterology 2005; 129(1): 42–49. 16012933 10.1053/j.gastro.2005.04.006

[10] Suares NC Ford AC. Prevalence of, and risk factors for, chronic idiopathic constipation in the community: systematic review and meta-analysis. Am J Gastroenterol 2011; 106(9): 1582–1591. 21606976 10.1038/ajg.2011.164

[11] Mugie SM Benninga MA Di Lorenzo C. Epidemiology of constipation in children and adults: a systematic review. Best Pract Res Clin Gastroenterol 2011; 25(1): 3–18. 21382575 10.1016/j.bpg.2010.12.010

[12] Peinado Molina RA Hernández Martínez A Martínez Vázquez S , et al. Influence of pelvic floor disorders on quality of life in women. Front Public Health 2023; 11: 1180907. 37942254 10.3389/fpubh.2023.1180907 PMC10629477

[13] Sung VW Washington B Raker CA. Costs of ambulatory care related to female pelvic floor disorders in the United States. Am J Obstet Gynecol 2010; 202(5): 483.e1–483.e4. 10.1016/j.ajog.2010.01.015 PMC2866792 20227673

[14] DeLancey JOL Kane Low L Miller JM , et al. Graphic integration of causal factors of pelvic floor disorders: an integrated life span model. Am J Obstet Gynecol 2008; 199(6): 610.e1–610.e5. 10.1016/j.ajog.2008.04.001 PMC2764236 18533115

[15] Loenneke JP Loprinzi PD Murphy CH , et al. Per meal dose and frequency of protein consumption is associated with lean mass and muscle performance. Clin Nutr 2016; 35(6): 1506–1511. 27086196 10.1016/j.clnu.2016.04.002

[16] Tanner SB Harwell SA. More than healthy bones: a review of vitamin D in muscle health. Ther Adv Musculoskelet Dis 2015; 7(4): 152–159. 26288665 10.1177/1759720X15588521 PMC4530385

[17] Smith GI Julliand S Reeds DN , et al. Fish oil–derived n−3 PUFA therapy increases muscle mass and function in healthy older adults1. Am J Clin Nutr 2015; 102(1): 115–122. 25994567 10.3945/ajcn.114.105833 PMC4480667

[18] Carvalhais A Araújo J Natal Jorge R , et al. Urinary incontinence and disordered eating in female elite athletes. J Sci Med Sport 2019; 22(2): 140–144. 30098973 10.1016/j.jsams.2018.07.008

[19] Rebullido TR Stracciolini A. Pelvic floor dysfunction in female athletes: is relative energy deficiency in sport a risk factor? Curr Sports Med Rep 2019; 18(7): 255–257. 31283625 10.1249/JSR.0000000000000615

[20] Miedel A Tegerstedt G Mæhle-Schmidt M , et al. Nonobstetric risk factors for symptomatic pelvic organ prolapse. Obstet Gynecol 2009; 113(5): 1089–1097. 19384125 10.1097/AOG.0b013e3181a11a85

[bibr21-17455057241305075] Subak LL Johnson C Johnson C , et al. Does weight loss improve incontinence in moderately obese women? Int Urogynecology J 2002; 13(1): 40–43. 10.1007/s001920200008 11999205

[bibr22-17455057241305075] Hannestad YS Rortveit G Daltveit AK , et al. Are smoking and other lifestyle factors associated with female urinary incontinence? The Norwegian EPINCONT Study. BJOG 2003; 110(3): 247–254. 12628262

[23] Hendrix SL Clark A Nygaard I , et al. Pelvic organ prolapse in the women’s health initiative: gravity and gravidity. Am J Obstet Gynecol 2002; 186(6): 1160–116. 10.1067/mob.2002.123819 12066091

[24] Drobnjak S Atsiz S Ditzen B , et al. Restrained eating and self-esteem in premenopausal and postmenopausal women. J Eat Disord 2014; 2(1): 23. 25349697 10.1186/s40337-014-0023-1 PMC4209048

[25] Baker JH Girdler SS Bulik CM. The role of reproductive hormones in the development and maintenance of eating disorders. Expert Rev Obstet Gynecol 2012; 7(6): 573–583. 23585773 10.1586/eog.12.54 PMC3622542

[bibr26-17455057241305075] Roney JR Simmons ZL. Ovarian hormone fluctuations predict within-cycle shifts in women’s food intake. Horm Behav 2017; 90: 8–14. 28202355 10.1016/j.yhbeh.2017.01.009

[27] Baker JH Runfola CD. Eating disorders in midlife women: a perimenopausal eating disorder? Maturitas 2016; 85: 112–116. 26857889 10.1016/j.maturitas.2015.12.017

[28] Laakkonen EK Kovanen V Sipilä S . Data from estrogenic regulation of muscle apoptosis (ERMA) study, 2022. https://jyx.jyu.fi/handle/123456789/83491 10.1097/GME.0000000000001117 PMC6110369 29738416

[29] Kovanen V Aukee P Kokko K , et al. Design and protocol of Estrogenic Regulation of Muscle Apoptosis (ERMA) study with 47 to 55-year-old women’s cohort: novel results show menopause-related differences in blood count. Menopause 2018; 25(9): 1020–1032. 29738416 10.1097/GME.0000000000001117 PMC6110369

[30] Cuschieri S. The STROBE guidelines. Saudi J Anaesth 2019; 13(Suppl 1): S31–S34. 10.4103/sja.SJA_543_18 PMC6398292 30930717

[31] Kuutti MA Hyvärinen M Kauppinen M , et al. Early adulthood and current physical activity and their association with symptoms of pelvic floor disorders in middle-aged women: an observational study with retrospective physical activity assessment. BJOG 2023; 130(6): 664–673. 36655435 10.1111/1471-0528.17397

[32] Keski-Rahkonen A Neale BM Bulik CM , et al. Intentional weight loss in young adults: sex-specific genetic and environmental effects. Obes Res 2005; 13(4): 745–753. 15897484 10.1038/oby.2005.84

[33] Masip G Keski-Rahkonen A Pietiläinen KH , et al. Development of a food-based diet quality score from a short FFQ and associations with obesity measures, eating styles and nutrient intakes in Finnish Twins. Nutrients 2019; 11(11): 2561. 31652865 10.3390/nu11112561 PMC6893528

[34] Juppi HK Sipilä S Cronin NJ , et al. Role of menopausal transition and physical activity in loss of lean and muscle mass: a follow-up study in middle-aged finnish Women. J Clin Med 2020; 9(5): 1588. 32456169 10.3390/jcm9051588 PMC7290663

[35] Nordic Nutrition Recommendations 2012: Integrating nutrition and physical activity. Nordisk Ministerråd [cited November 8, 2024], https://urn.kb.se/resolve?urn=urn:nbn:se:norden:org:diva-2561 (2014).

[36] Hirvensalo M Lintunen T Rantanen T. The continuity of physical activity—a retrospective and prospective study among older people. Scand J Med Sci Sports 2000; 10(1): 37–41. 10693611 10.1034/j.1600-0838.2000.010001037.x

[37] Kujala UM Kaprio J Sarna S , et al. Relationship of leisure-time physical activity and mortality: the Finnish Twin cohort. JAMA 1998; 279(6): 440–444. 9466636 10.1001/jama.279.6.440

[38] Harlow SD Gass M Hall JE , et al. Executive summary of the Stages of Reproductive Aging Workshop 10: addressing the unfinished agenda of staging reproductive aging. Menopause 2012; 19(4): 387. 22343510 10.1097/gme.0b013e31824d8f40 PMC3340903

[39] van Buuren S . Mice: multivariate imputation by chained equations in R. J Stat Softw 2011; 45(3): 1–67.

[40] R Core Team. R: a language and environment for statistical computing. Vienna, Austria: R Foundation for Statistical Computing, 2024.

[41] Slevec JH Tiggemann M. Predictors of body dissatisfaction and disordered eating in middle-aged women. Clin Psychol Rev 2011; 31(4): 515–524. 21239098 10.1016/j.cpr.2010.12.002

[42] Stice E. Risk and maintenance factors for eating pathology: a meta-analytic review. Psychol Bull 2002; 128(5): 825–848. 12206196 10.1037/0033-2909.128.5.825

[43] Mangweth-Matzek B Hoek HW Rupp CI , et al. The menopausal transition—a possible window of vulnerability for eating pathology. Int J Eat Disord 2013; 46(6): 609–616. 23847142 10.1002/eat.22157

[44] Thompson KA Bardone-Cone AM. Menopausal status and disordered eating and body image concerns among middle-aged women. Int J Eat Disord 2019; 52(3): 314–318. 30702172 10.1002/eat.23030

[45] Baker JH Peterson CM Thornton LM , et al. Reproductive and appetite hormones and bulimic symptoms during midlife. Eur Eat Disord Rev 2017; 25(3): 188–194. 28276114 10.1002/erv.2510 PMC5421373

[46] Mangweth-Matzek B Rupp CI Vedova S , et al. Disorders of eating and body image during the menopausal transition: associations with menopausal stage and with menopausal symptomatology. Eat Weight Disord 2021; 26(8): 2763–2769. 33595812 10.1007/s40519-021-01141-4

[47] Butera PC. Estradiol and the control of food intake. Physiol Behav 2010; 99(2): 175–180. 19555704 10.1016/j.physbeh.2009.06.010 PMC2813989

[bibr48-17455057241305075] Eckel LA. The ovarian hormone estradiol plays a crucial role in the control of food intake in females. Physiol Behav 2011; 104(4): 517–524. 21530561 10.1016/j.physbeh.2011.04.014 PMC3139826

[bibr49-17455057241305075] Brown LM Clegg DJ. Central effects of estradiol in the regulation of food intake, body weight, and adiposity. J Steroid Biochem Mol Biol 2010; 122(1): 65–73. 20035866 10.1016/j.jsbmb.2009.12.005 PMC2889220

[bibr50-17455057241305075] Asarian L Geary N. Modulation of appetite by gonadal steroid hormones. Philos Trans R Soc B Biol Sci 2006; 361(1471): 1251–1263. 10.1098/rstb.2006.1860 PMC1642706 16815802

[51] Chai JK Blaha V Meguid MM , et al. Use of orchiectomy and testosterone replacement to explore meal number-to-meal size relationship in male rats. Am J Physiol Regul Integr Comp Physiol 1999; 276(5): R1366–R1373. 10.1152/ajpregu.1999.276.5.R1366 10233029

[52] Eckel LA. Estradiol: a rhythmic, inhibitory, indirect control of meal size. Physiol Behav 2004; 82(1): 35–41. 15234587 10.1016/j.physbeh.2004.04.023

[53] Wadden TA Brownell KD Foster GD. Obesity: responding to the global epidemic. J Consult Clin Psychol 2002; 70(3): 510–525. 12090366 10.1037//0022-006x.70.3.510

[54] Herman CP Mack D. Restrained and unrestrained eating1. J Pers 1975; 43(4): 647–660. 1206453 10.1111/j.1467-6494.1975.tb00727.x

[55] Copeland AL Martin PD Geiselman PJ , et al. Predictors of pretreatment attrition from smoking cessation among pre- and postmenopausal, weight-concerned women. Eat Behav 2006; 7(3): 243–251. 16843227 10.1016/j.eatbeh.2005.10.001

[56] Adams RC Sedgmond J Maizey L , et al. Food addiction: implications for the diagnosis and treatment of overeating. Nutrients 2019; 11(9): 2086. 31487791 10.3390/nu11092086 PMC6770567

[57] Hayes JF Schumacher LM Panza E , et al. Affective responses to overeating episodes in women participating in a behavioral weight loss program. Eat Behav 2022; 44: 101599. 35144169 10.1016/j.eatbeh.2022.101599 PMC8901183

[58] Christian C Perko VL Vanzhula IA , et al. Eating disorder core symptoms and symptom pathways across developmental stages: a network analysis. J Abnorm Psychol 2020; 129(2): 177–190. 31714097 10.1037/abn0000477

[59] Brown TA Forney KJ Klein KM , et al. A 30-year longitudinal study of body weight, dieting, and eating pathology across women and men from late adolescence to later mid-life. J Abnorm Psychol 2020; 129(4): 376–386. 32309984 10.1037/abn0000519 PMC7179079

[60] Dallosso H Matthews R McGrother C , et al. Diet as a risk factor for the development of stress urinary incontinence: a longitudinal study in women. Eur J Clin Nutr 2004; 58(6): 920–926. 15164113 10.1038/sj.ejcn.1601913

[61] Maserejian NN Giovannucci EL McVary KT , et al. Dietary macronutrient and energy intake and urinary incontinence in women. Am J Epidemiol 2010; 171(10): 1116–1125. 20421220 10.1093/aje/kwq065 PMC2877472

[62] Kim D Hou W Wang F , et al. Factors affecting obesity and waist circumference among US adults. Prev Chronic Dis 2019; 16: E02. 10.5888/pcd16.180220 PMC6341820 30605422

[63] Drewnowski A Kurth C Holden-Wiltse J , et al. Food preferences in human obesity: carbohydrates versus fats [cited June 6, 2024], http://deepblue.lib.umich.edu/handle/2027.42/30004 (1992). 10.1016/0195-6663(92)90198-f 1510463

[64] Hunskaar S. Epidemiology and natural history of urinary incontinence. Int Urogynecology J 2000; 11(5): 301–319. 10.1007/s001920070021 11052566

[65] Malesza IJ Malesza M Walkowiak J , et al. High-fat, Western-style diet, systemic inflammation, and gut microbiota: a narrative review. Cells 2021; 10(11): 3164. 34831387 10.3390/cells10113164 PMC8619527

[66] Post WM Widomska J Grens H , et al. Molecular processes in stress urinary incontinence: a systematic review of human and animal studies. Int J Mol Sci 2022; 23(6): 3401. 35328824 10.3390/ijms23063401 PMC8949972

[67] Marcelissen T Anding R Averbeck M , et al. Exploring the relation between obesity and urinary incontinence: Pathophysiology, clinical implications, and the effect of weight reduction, ICI-RS 2018. Neurourol Urodyn 2019; 38(Suppl 5): S18–S24. 10.1002/nau.24072 31821633

[68] Kupelian V McVary KT Barry MJ , et al. Association of C-reactive protein and lower urinary tract symptoms in men and women. Results from the Boston Area Community Health (BACH) survey. Urology 2009; 73(5): 950–957. 19394490 10.1016/j.urology.2008.12.012 PMC3551271

[69] Majdan M Bobrowska-Korczak B. Active compounds in fruits and inflammation in the body. Nutrients 2022; 14(12): 2496. 35745226 10.3390/nu14122496 PMC9229651

[70] Jura YH Townsend MK Curhan GC , et al. Caffeine intake and risk of stress, urgency, and mixed urinary incontinence. J Urol 2011; 185(5): 1775–1780. 21420114 10.1016/j.juro.2011.01.003 PMC3077934

[bibr71-17455057241305075] Raju R Linder BJ. Evaluation and treatment of overactive bladder in women. Mayo Clin Proc 2020; 95(2): 370–377. 32029089 10.1016/j.mayocp.2019.11.024

[72] Townsend MK Jura YH Curhan GC , et al. Fluid intake and risk of stress, urgency, and mixed urinary incontinence. Am J Obstet Gynecol 2011; 205(1): 73.e1–73.e6. 10.1016/j.ajog.2011.02.054 PMC3135667 21481835

[73] Eoff JC. Optimal treatment of chronic constipation in managed care: review and roundtable discussion. J Manag Care Pharm 2008; 14(9 Suppl A): 1–15. 18950252 10.18553/jmcp.2008.14.S8-A.1 PMC10438150

[74] Suares NC Ford AC. Systematic review: the effects of fibre in the management of chronic idiopathic constipation. Aliment Pharmacol Ther 2011; 33(8): 895–901. 21332763 10.1111/j.1365-2036.2011.04602.x

[75] Dukas L Willett WC Giovannucci EL. Association between physical activity, fiber intake, and other lifestyle variables and constipation in a study of women. Am J Gastroenterol 2003; 98(8): 1790–1796. 12907334 10.1111/j.1572-0241.2003.07591.x

[76] Müller-lissner SA Kamm MA Scarpignato C , et al. Myths and misconceptions about chronic constipation. Am J Gastroenterol 2005; 100(1): 232–242. 15654804 10.1111/j.1572-0241.2005.40885.x

[77] Andy UU Ejike N Khanijow KD , et al. Diet modifications in older women with fecal incontinence: a qualitative study. Female Pelvic Med Reconstr Surg 2020; 26(4): 239–243. 30747728 10.1097/SPV.0000000000000702 PMC6687572

[78] Colavita K Andy UU. Role of diet in fecal incontinence: a systematic review of the literature. Int Urogynecology J 2016; 27(12): 1805–1810. 10.1007/s00192-016-2979-7 26883367

[79] Croswell E Bliss DZ Savik K. Diet and eating pattern modifications used by community living adults to manage their fecal incontinence. J Wound Ostomy Cont Nurs 2010; 37(6): 677–682. 10.1097/WON.0b013e3181feb017 PMC2994718 21076267

[80] Markland AD Richter HE Burgio KL , et al. Fecal incontinence in obese women with urinary incontinence: prevalence and role of dietary fiber intake. Am J Obstet Gynecol 2009; 200(5): 566.e1–566.e6. 10.1016/j.ajog.2008.11.019 PMC2670959 19136088

[81] Halverson AL. Nonoperative management of fecal incontinence. Clin Colon Rectal Surg 2005; 18(1): 17–21. 20011335 10.1055/s-2005-864077 PMC2780124

[82] Chang J McLemore E Tejirian T. Anal health care basics. Perm J 2016; 20(4): 15–222. 10.7812/TPP/15-222 PMC5101094 27723447

[83] Mortel TF van de . Faking it: social desirability response bias in self-report research. Aust J Adv Nurs Online 2008; 25(4): 40–42, 45–48.

[84] Keski-Rahkonen A Bulik CM Pietiläinen KH , et al. Eating styles, overweight and obesity in young adult twins. Eur J Clin Nutr 2007; 61(7): 822–829. 17251930 10.1038/sj.ejcn.1602601

[85] Mossavar-Rahmani Y Tinker LF Huang Y , et al. Factors relating to eating style, social desirability, body image and eating meals at home increase the precision of calibration equations correcting self-report measures of diet using recovery biomarkers: findings from the Women’s Health Initiative. Nutr J 2013; 12(1): 63. 23679960 10.1186/1475-2891-12-63 PMC3658913

[86] Adams SA Matthews CE Ebbeling CB , et al. The effect of social desirability and social approval on self-reports of physical activity. Am J Epidemiol 2005; 161(4): 389–398. 15692083 10.1093/aje/kwi054 PMC2958515

