# Supplementary material for: Association of eating behavior with symptoms of pelvic floor disorders in middle-aged women: An observational study
Source: Womens Health (Lond). 2024 Dec 10;20:17455057241305075. doi: 10.1177/17455057241305075 (PMC11632885; doi:10.1177/17455057241305075)
Supplement: sj-docx-1-whe-10.1177_17455057241305075 – Supplemental material for Association of eating behavior with symptoms of pelvic floor disorders in middle-aged women: An observational study [file sj-docx-1-whe-10.1177_17455057241305075.docx]

**Appendix 1.** Pelvic floor disorder questionnaire

Pelvic floor dysfunction (based on your own experience or evaluation)

|  | Yes  (x) | No  (x) | Since when have you had the symptom? (year) |
| --- | --- | --- | --- |
| Have you had within the last month urinary incontinence during physical effort or coughing? |  |  |  |
| Have you had within the last month urge or urgency-related urinary incontinence? |  |  |  |
| Have you had within the last month fecal incontinence? |  |  |  |
| Have you had within the last month constipation or defecation difficulties? |  |  |  |
| Have you had within the last month a feeling that something is falling out of your vagina? |  |  |  |
