# Supplementary material for: Association of eating behavior with symptoms of pelvic floor disorders in middle-aged women: An observational study
Source: Womens Health (Lond). 2024 Dec 10;20:17455057241305075. doi: 10.1177/17455057241305075 (PMC11632885; doi:10.1177/17455057241305075)
Supplement: sj-docx-2-whe-10.1177_17455057241305075 – Supplemental material for Association of eating behavior with symptoms of pelvic floor disorders in middle-aged women: An observational study [file sj-docx-2-whe-10.1177_17455057241305075.docx]

**Appendix 2.** Pelvic floor disorders in total analytical sample and in participants with different types of eating style (n=1098)

|  | **Total analytical sample (*n*=1098)** | **Normal eating**  **(*n*= 589)** | **Overeating (*n*=356)** | **Restrictive eating (*n*=116)** | **Alternating overeating and restricting (*n*=35)** | **Eating styles missing data (*n*=2)** |
| --- | --- | --- | --- | --- | --- | --- |
| **Stress urinary incontinence** |  |  |  |  |  |  |
| yes | 440 (40.3) | 208 (35.5) | 159 (45.0) | 55 (47.4) | 17 (48.6) | 1 |
| no | 652 (59.7) | 378 (64.5) | 194 (55.0) | 61 (52.6) | 18 (51.4) | 1 |
| missing data, *n* | 6 | 3 | 3 | 0 | 0 |  |
| **Urgency urinary incontinence** |  |  |  |  |  |  |
| yes | 149 (13.6) | 76 (12.9) | 48 (13.6) | 18 (15.5) | 7 (20.0) | 0 |
| no | 943 (86.4) | 511 (87.1) | 304 (86.4) | 98 (84.5) | 28 (80.0) | 2 |
| missing data, *n* | 6 | 2 | 4 | 0 | 0 |  |
| **Fecal incontinence** |  |  |  |  |  |  |
| yes | 34 (3.1) | 13 (2.2) | 13 (3.7) | 6 (5.2) | 2 (5.9) | 0 |
| no | 1056 (96.9) | 573 (97.8) | 340 (96.3) | 109 (94.8) | 32 (94.1) | 2 |
| missing data, *n* | 8 | 3 | 3 | 1 | 1 |  |
| **Constipation or defecation difficulties** |  |  |  |  |  |  |
| yes | 189 (17.3) | 88 (15.0) | 64 (18.1) | 29 (25.2) | 8 (22.9) | 0 |
| no | 903 (82.7) | 499 (85.0) | 289 (81.9) | 86 (74.8) | 27 (77.1) | 2 |
| missing data, *n* | 6 | 2 | 3 | 1 | 0 |  |
